# Culture-adapted *Plasmodium falciparum* isolates from UK travellers: *in vitro* drug sensitivity, clonality and drug resistance markers

**DOI:** 10.1186/1475-2875-12-320

**Published:** 2013-09-13

**Authors:** Donelly A van Schalkwyk, Rebekah Burrow, Gisela Henriques, Nahla B Gadalla, Khalid B Beshir, Christian Hasford, Stephen G Wright, Xavier C Ding, Peter L Chiodini, Colin J Sutherland

**Affiliations:** 1 Faculty of Infectious and Tropical Diseases, London School of Hygiene and Tropical Medicine, Keppel Street, London WC1E 7HT, UK; 2 Department for Acute Medicine and Chest Medicine, University College London Hospitals, London NW1 2PG, UK; 3 Department of Clinical Parasitology, Hospital for Tropical Diseases, Mortimer Market Centre, Capper Street, London WC1E 6JB, UK; 4 Medicines for Malaria Venture, 20 rte de Pré Bois, Geneva CH 1215, Switzerland

## Abstract

**Background:**

The screening of lead compounds against *in vitro* parasite cultures is an essential step in the development of novel anti-malarial drugs, but currently relies on laboratory parasite lines established *in vitro* during the last century. This study sought to establish in continuous culture a series of recent *Plasmodium falciparum* isolates to represent the current parasite populations in Africa, all of which are now exposed to artemisinin combination therapy.

**Methods:**

Pre-treatment *P. falciparum* isolates were obtained in EDTA, and placed into continuous culture after sampling of DNA. One post-treatment blood sample was also collected for each donor to monitor parasite clonality during clearance *in vivo*. IC_50_ estimates were obtained for 11 anti-malarial compounds for each established parasite line, clonal multiplicity measured *in vivo* and *in vitro*, and polymorphic sites implicated in parasite sensitivity to drugs were investigated at the *pfmdr1*, *pfcrt*, *pfdhfr, pfdhps* and *pfap2mu* loci before and after treatment, and in the cultured lines.

**Results:**

*Plasmodium falciparum* isolates from seven malaria patients with recent travel to three West African and two East African countries were successfully established in long-term culture. One of these, HL1211, was from a patient with recrudescent parasitaemia 14 days after a full course of artemether-lumefantrine. All established culture lines were shown to be polyclonal, reflecting the *in vivo* isolates from which they were derived, and at least two lines reliably produce gametocytes *in vitro*. Two lines displayed high chloroquine IC_50_ estimates, and carried the CVIET haplotype at codons 72–76, whereas the remaining five lines carried the CVMNK haplotype and were sensitive *in vitro*. All were sensitive to the endoperoxides dihydroartemisinin and OZ277, but IC_50_ estimates for lumefantrine varied, with the least sensitive parasites carrying *pfmdr1* alleles encoding Asn at codon 86.

**Conclusions:**

This study describes the establishment in continuous culture, *in vitro* drug sensitivity testing and molecular characterization of a series of multiclonal *P. falciparum* isolates taken directly from UK malaria patients following recent travel to various malaria-endemic countries in Africa. These “HL” isolates are available as an open resource for studies of drug response, antigenic diversity and other aspects of parasite biology.

## Background

There are currently six species of the genus *Plasmodium* known to infect humans: *Plasmodium falciparum*, *Plasmodium vivax*, *Plasmodium ovale curtisi*, *Plasmodium ovale wallikeri*, *Plasmodium malariae* and *Plasmodium knowlesi*[1,2]. Of these, *P. falciparum* is the species responsible for most of the mortality and morbidity associated with the disease and it is during the asexual intra-erythrocytic stages that most of the symptoms of malaria are manifest. Studies into many aspects of human malaria parasite biology were greatly advanced by the development of a method to culture asexual blood stages of *P. falciparum in vitro* in 1976 [3]. Since then, several parasite strains and clones have been cultivated from varying geographical regions, allowing researchers to explore differences in parasite phenotypes as diverse as immune evasion in mosquitoes [4] and red cell invasion [5] to the development of drug resistance [6,7]. However, the most widely studied *in vitro* parasite strains or clones have been in use for more than two decades (Table 1) and therefore pre-date the era of widespread artemisinin-based combination therapy (ACT) implementation. In addition, many of these parasite lines originate from Asia or the Americas (Table 1). However, it is in Africa where the majority of deaths from malaria are reported, and the greatest transmission intensity occurs [8]. Moreover, information about the infection from which the parasites lines were isolated (i e, possible parasite drug exposure from prophylaxis, patient travel histories, etc.) is not easy to access, if at all available.

**Table 1:** **List of *Plasmodium falciparum *strains/clones in current widespread use for *in vitro *studies**

**Strain/Clone**^**§**^	**Origin**	**Region**	Year reported*	**Reference**
**Chloroquine sensitive**				
NF-54	Netherlands	Europe	1981	[9,10]
3D7 (cloned from NF-54)	Netherlands	Europe	1987	[11]
D10 (cloned from FC27)	Papua New Guinea	Oceania	1983	[12]
HB3 (cloned from Honduras I/CDC)	Honduras	Central America	1984	[13]
D6 (cloned from Sierra Leone I/CDC)	Sierra Leone	Africa	1988	[14]
T9-96 (cloned from T9)	Thailand	Asia	1981	[15,16]
GB4 (cloned from Ghana III/CDC)	Ghana	Africa	2003	[17]
ITG2F6 (cloned from Ituxi 084)	Brazil	South America	1979	[18]
**Chloroquine resistant**				
K1	Thailand	Asia	1981	[19]
FCR3	The Gambia	Africa	1981	[20]
W2 (cloned from Indo III/CDC)	Indochina	Asia	1988	[14]
W2mef (derived from W2)	Indochina	Asia	1988	[21]
Dd2 (cloned from W2mef)	Indochina	Asia	1988	[22]
7G8 (cloned from IMTM22)	Brazil	South America	1984	[23]
V1/S (cloned from V1)	Vietnam	Asia	1990	[24]
Malayan Camp	Malaya	Asia	1965	[25]

^**§**^This list is not meant to reflect an exhaustive reference of all the parasite lines in use world-wide and it excludes the multiple progeny of the HB3 × Dd2 genetic cross or the 7G8 × GB4 genetic cross. Instead, it represents a list of parasites that predominate in the literature still today in several genetic and drug sensitivity studies.

*These are the earliest literature reports for the parasite lines/clones listed so the actual date of establishment in culture pre-dates these references in each case.

Since 2001 the World Health Organization (WHO) has recommended ACT as first-line treatment for uncomplicated *P. falciparum* malaria infections [26]. ACT involves pairing an artemisinin derivative, with its short elimination half-life, with a longer-lasting anti-malarial partner drug (e g, artemether-lumefantrine, dihydroartemisinin-piperaquine, etc.). This approach to treatment has been highly successful for several years in areas where drug resistance to chloroquine or sulphadoxine-pyrimethamine had been widespread [27]. However, an increasing number of infections exhibiting reduced sensitivity to artemisinin *in vivo* are being observed in areas of western Cambodia, Thailand and recently southern Myanmar [28-32]. This has been attributed to a delayed clearance phenotype with recent evidence suggesting that parasites have developed a mechanism that allows them to remain dormant in the ring stage of their life cycle in order to evade the toxic effects of the short half-life artemisinin derivative [33,34]. This developing altered phenotype highlights a need to update *in vitro* parasite lines in order to study parasites whose behaviour reflect the current anti-malarial exposure patterns.

There are currently no validated molecular markers for parasites with reduced susceptibility to artemisinin, but persistent parasitaemia after treatment with artemether-lumefantrine has been associated with particular alleles of *pfmdr1 in vivo*[35]. Recent published data from studies of artemisinin resistance in the rodent parasite *Plasmodium chabaudi* has identified a new candidate locus*, pcap2mu*, encoding the clathrin-associated μ adaptor protein 2 [36]. These authors described potentially important sequence diversity in the *P. falciparum* homologue, *pfap2mu* (PF3D7_1218300), between codons 146 and 437, in a series of clinical isolates.

The aim of this study was to establish a fully characterized panel of recent *P. falciparum* isolates, adapted to grow under standard *in vitro* culture conditions, for studies of sensitivity to both established and investigational anti-malarial drugs. This small series of newly culture-adapted *P. falciparum* isolates, together with relevant genotyping analysis at loci of interest and drug response phenotypes, has been made available as a resource for researchers wishing to work *in vitro* with African parasites from the current ACT era.

## Methods

### Patient sample acquisition

Voluntary consent and travel history was obtained from patients presenting with malaria to the Hospital for Tropical Diseases, London (HTD), or the Accident and Emergency Department of University College London Hospitals (UCLH). A venous blood sample was obtained in a 4 mL Vacutainer tube containing EDTA (BD, Oxfordshire, UK). A few drops of blood were removed to prepare thick and thin blood smears in order to confirm diagnosis and to establish parasitaemia. A 200 μL aliquot of whole blood was removed for DNA extraction for use in later genotyping assays. The remaining sample was then used for the laboratory adaptation of the malaria parasites in the malaria culture laboratories, London School of Hygiene & Tropical Medicine (LSHTM). All patient identifiers were removed and a code assigned to each parasite sample. Approval for the study was obtained from the Research Ethics Committee of the University College London Hospitals (Application number: 07/Q0505/60). All seven patients described herein were managed as in-patients, with fully observed antimalarial treatment. Those treated with AL received the standard dosage of Riamet© as recommended by the manufacturer (Novartis Pharmaceuticals UK Ltd), which comprises six doses of four tablets each, taken over 60 hours. Each tablet contains 20 mg artemether and 120 mg lumefantrine.

### General reagents and compounds for growth inhibition studies

Albumax II and gentamicin sulphate were purchased from Invitrogen (Carlsbad, CA, USA). HEPES, RPMI 1640, human AB serum, hypoxanthine, L-glutamine and D-glucose were purchased from Sigma-Aldrich (St Louis, MO, USA). Anti-malarial compounds/drugs used in this study were supplied by the Medicines for Malaria Venture (MMV, Geneva, Switzerland). Primary stock solutions for all compounds, except chloroquine, were first made by dissolution in dimethyl sulphoxide (DMSO). Subsequent dilutions were made in complete RPMI growth media such that the amount of DMSO present in the test concentration range was non-toxic to parasites. Chloroquine primary stocks were made by dissolution in deionized water (Millipore, Watford, UK).

### Laboratory adaptation and parasite culture

The whole blood sample obtained from the patients was first washed twice in RPMI growth media in order to separate the red cells from the plasma. Briefly, the whole blood was suspended in 6 mL of RPMI media and centrifuged at 2,000 × g for 10 min. The supernatant and buffy coat were then removed and the red cell pellet was suspended in approximately 8 mL of RPMI before a second centrifugation step as described above. After removing the supernatant the parasitized red cells were resuspended in complete medium (CM) for culture adaptation. For the first isolate obtained (HL1204) this was composed of RPMI 1640 growth medium supplemented with 10% (v/v) human AB serum, 25 mg/L gentamicin, 147 μM hypoxanthine, 25 mM HEPES, and 25 mM Na_2_HCO_3_. For all subsequent isolate adaptation and routine parasite cultivation the amount of human serum added was reduced to 2% (v/v) and the CM further supplemented with 5 g/L Albumax II, 10 mM D-glucose and 2 mM L-glutamine. The parasites were maintained in continuous culture with shaking. The parasites were stored in a sealed flask incubated at 37°C under a gas mixture of 93% N_2_, 4% CO_2_ and 3% O_2_. As the parasitaemia increased the parasites were diluted in human A^+^ blood (National Health Blood & Transplant, UK) to maintain a parasitaemia <4% and a haematocrit of 4%.

Early passage stabilates of all seven lines have been deposited at the European Malaria Reagent Repository [37] and are available for general research use.

### Anti-plasmodial activity assays

Growth curves were initiated on ring stage parasites with a final parasitaemia of 0.5% and a haematocrit of 2% in a 200 μL volume within 96-well microtitre plates. Serial two- or three-fold drug dilutions were first performed across the plate in duplicate wells and the parasites added thereafter. The plate was incubated for 48 hrs at 37°C and parasite growth was measured using the PicoGreen method [38]. Briefly, PicoGreen (Invitrogen, P7581) was diluted 1:200 into a lysis buffer made up of 20 mM Tris, 5 mM EDTA, 0.008% (w/v) saponin and 0.08% Triton X-100 (pH 7.5). Thereafter, 50 μL of this solution was pipetted into each well of a duplicate 96-well plate. Then 100 μL of the resuspended parasite solution was transferred from the original growth assay plate into the Picogreen plate and was mixed well. This plate was kept in the dark for at least 10 min and was read in a Spectramax M3 microplate reader (Molecular Devices, Berkshire, UK) at an excitation wavelength of 485 nm and an emission wavelength of 538 nm.

### DNA isolation

Parasite genomic DNA was extracted from 200 μL of whole blood (for patient-derived samples) or 200 μL of packed red cells (for culture-adapted parasites) using the QIAamp DNA Blood Mini Kit (Qiagen, UK) as per the manufacturer’s instructions. The DNA was stored at −80°C.

### Estimating multiplicity of infection

The *P. falciparum* isolates were assessed for multiplicity of infection (MOI) by nested PCR analysis of *msp1* block 2 (K1, MAD20, RO33) and *msp2* block 3 (FC27, 3D7/IC). These alleles are characterized by conserved regions flanked by repeat sequences of variable length; size variation within the alleles can be used to discriminate different parasite clones by PCR fragment length polymorphism. Previously designed primer sets were used but with modified cycling conditions [39], as follows. Nest 1 amplifications were performed in 25 μL reaction mixture containing 5 μL DNA, NH_4_ buffer, 4 mM MgCl_2_, 1 mM deoxynucleotides (dNTPs), 0.2 μM forward primer, 0.2 μM reverse primer and 1 unit of BIOTAQ™ (Bioline, UK). The cycling conditions were: denaturation at 94°C for 3 min, 30 cycles of 94°C for 30 sec, 58°C for 1 min and 68°C for 1 min, followed by a final extension of 5 min at 68°C.

Nest 2 amplifications were performed in a 25 μL reaction mixture containing 1 μL of nest 1 product diluted 1/10, NH_4_ buffer, 4 mM MgCl_2_, 1 mM dNTPs, 0.2 μM forward primer, 0.2 μM reverse primer and 1 unit of BIOTAQ. The cycling conditions were: denaturation at 94°C for 3 min, 30 cycles of 94°C for 30 sec, 61°C for 1 min and 72°C for 1 min, followed by a final extension of 5 min at 72°C.

In order to separate and visualize the amplification products by electrophoresis, 15 μL of amplification products mixed with loading buffer was loaded onto 2% agarose gels in 0.5% TBE buffer, stained with ethidium bromide (EtBr). The minimum MOI was calculated to be the highest observed number of size variants for either *pfmsp1* or *pfmsp2* for any sample (Day 0, Day 1, culture-adapted) of an isolate.

### Genotyping of drug resistance markers

#### pfcrt

The genotype for *pfcrt* was determined by multiplex real-time qPCR performed in a Rotorgene RG3000 thermocycler (Corbett Research, Australia), in which a small segment around codons 72–76 was amplified in the presence of three dual-labelled probes complementary to CVMNK (FAM fluorophore), CVIET (JOE fluorophore) and SVMNT (ROX fluorophore) exactly as described previously [40,41]. For data analysis, a threshold for each probe was set manually using the positive and negative controls.

#### pfmdr1

Amplification of *pfmdr1* for sequencing required two amplicons, as described previously [42], with minor modifications as follows [see Additional file 1]. For fragment 1, the nest 1 25 μL reaction mixture contained 5 μL DNA, NH_4_ buffer, 2 mM MgCl_2_, 1 mM dNTPs, 0.2 μM of each primer and 1 unit of BIOTAQ. The cycling conditions were 94°C for 3 min followed by 30 cycles of 94°C for 30 sec, 55°C for 30 sec, 65°C for 1 min, and then a final elongation at 65°C for 5 min. The nest 2 reaction contained 1 μL nest 1 product, primers MDR2/1 and NEWREV1 and other reagents as for the nest 1 reaction. Cycling conditions were 94°C for 3 min followed by 30 cycles of 94°C for 30 sec, 60°C for 30 sec, 65°C for 1 min, and then a final elongation at 65°C for 5 min. For fragment 2, the nest 1 25 μL reaction mixture contained 5 μL DNA, NH_4_ buffer, 2 mM MgCl_2_, 1 mM dNTPs, 0.2 μM of each primer and 1 unit of BIOTAQ. The cycling conditions were 94°C for 3 min followed by 35 cycles of 94°C for 30 sec, 55°C for 30 sec, 68°C for 45 sec, and then a final elongation at 68°C for 2 min. The nest 2 reaction contained 1 μL of nest 1 product, primers newfr2_F and newfr2_N2R and other reagents as for the nest 1 reaction. The cycling conditions were 94°C for 3 min followed by 35 cycles of 94°C for 30 sec, 55°C for 30 sec, 68°C for 45 sec, and then a final elongation at 68°C for 2 min. Amplicons were sequenced using BigDye Terminator v3.1 cycle sequencing kits and analysis on an ABI 3730 sequencer (Applied Biosystems). The sequences were analyzed using Geneious R6 software (Biomatters, New Zealand). Gene copy number for *pfmdr1* was determined by real-time qPCR using the DD-C_T_ method, exactly as described previously [35].

#### pfdhfr

Amplification of *pfdhfr* was performed by nested PCR amplification exactly as described elsewhere [43].

#### pfdhps

Amplification of *pfdhps* was performed by nested PCR amplification and partial sequencing using the amplification primers R2 and R/ described by Pearce *et al.*[43], with the following modifications. Each 25 μL reaction mixture contained 5 μL DNA, KCl buffer, 0.2 mM dNTPs, 0.4 μM of each primer and 1 unit of BIOTAQ. The cycling conditions were 93°C for 5 min followed by 40 cycles of 93°C for 20 sec, 55°C for 30 sec, 68°C for 75 sec, and then a final elongation at 68°C for 5 min. The nest 2 reaction contained 1 μL of nest 1 product, 0.4 μM each of primers midFWD and midREV [44] and other reagents as for the nest 1 reaction. The cycling conditions were 93°C for 5 minutes followed by 30 cycles of 93°C for 20 sec, 56°C for 30 sec, 68°C for 75 sec, and then a final elongation at 72°C for 5 min.

#### pfap2mu

Three nested fragments of *pfap2mu* (gene ID PF3D7_1218300) were amplified from a single primary amplicon as described [Hallett RL, Henriques G, Beshir KB, Gadalla NB, Burrow R, van Schalkwyk DA, Sawa P, Bousema T, Sutherland CJ: Sub-microscopic *Plasmodium falciparum* persisting in Kenyan children immediately after ACT show directional selection at the *pfmdr1*, *pfcrt*, *pfubp-1* and *pfap2mu* loci, in preparation.], using primers and conditions provided in the additional file [see Additional file 1]. Each amplification reaction was performed in a total volume of 25 μL with the following reaction mixture: 0.2 μM of each primer, 4.0 mM MgCl_2_, 0.4 mM dNTPs and 1 U Bioline Taq polymerase (Bioline, UK). Extracted DNA (5 μL) was added to each first round PCR mixture. One microliter of the first round product was then used as a template in a 25 μL nested amplification. The thermal cycle programme for the primary amplification was 94°C for 3 min, and 30 cycles of 94°C for 30 sec, annealing temperature for 30 sec and 68°C for 1 min with a final extension of 68°C for 15 min. The second round of PCR consisted of one initial denaturation hold at 94°C for 3 min, followed by 40 cycles of 94°C for 30 sec, annealing temperature for 30 sec and 68°C for 45 sec with a final extension of 68°C for 10 min.

### Sequencing of PCR products

PCR-amplified products from all loci were purified with Exonuclease I (ExoI) and Thermosensitive Alkaline Phosphatase (AP) enzymes (Fermentas, UK) in order to eliminate non-incorporated dNTPs and primers. The enzymatic purification was carried out in a final volume of 10 μL containing 5 μL of the PCR product, 3 U ExoI, 1 U AP and 1X AP reaction buffer, which were incubated for 60 min at 37°C followed by 15 min at 72°C for enzyme denaturation.

The cycle sequencing reactions were carried out using 1 μL ExoI/AP purified PCR amplicons, 0.5 μL BigDye^®^ Terminator v3.1 Cycle Sequencing Kit reaction mix (Applied Biosystems, UK), 0.2 μM of each PCR primer, 1.75 μL Big Dye Sequencing Buffer (Applied Biosystems) in a total volume of 10 μL. Single-base extension was performed as follows: one denaturation hold at 96°C for 1 min followed by 25 cycles of 96°C for 30 sec, 50°C for 15 sec, and 60°C for 4 min.

The sequenced reaction fragments were purified using ethanol/sodium acetate precipitation method, where 3 μL 3 NaOAc (pH 4.6), 62.5 μL 100% ethanol and 24.5 μL of nuclease free water were added to each sample. The samples were mixed by inversion and incubated at −20°C for 25 min. After incubation, samples were centrifuged at 4°C at 3000 g for 30 min. The supernatant was discarded by inversion, 150 μL ice cold 70% ethanol was added and the samples centrifuged at 4°C at 3000 g for 10 min. The supernatant was discarded by inversion and each sample was allowed to dry at room temperature for 30 min. Finally, pellets were resuspended in 10 μL Hi-Di formamide. Samples were fractionated on an ABI prism 3730 Genetic Analyzer (Applied Biosystems). Raw sequence data in the form of chromatograms from both sense and antisense primers were edited, aligned and assembled into contigs using Geneious Pro software (version 5.5.3; Biomatters, New Zealand), after resolving potential ambiguities by eye. Genetic polymorphisms were identified by comparing each sequence to that of the 3D7 reference genome.

### Statistics

*Plasmodium falciparum* sensitivity to anti-malarial compounds *in vitro* was calculated relative to untreated control wells of the same parasite line by first subtracting the background fluorescence (i e, from parasites exposed to a supralethal 10 μM chloroquine concentration) from all the remaining parasite-containing wells. The fluorescence signal from each drug-exposed parasite well was then divided by the average fluorescent signal from drug-free parasite control wells and converted to percentage viability. Viability *vs* concentration was plotted in SigmaPlot 2000 or Prism v4.02 and curves generated using sigmoidal dose response functions. The IC_50_ values were then extrapolated from these curves for each drug. Statistical comparisons of the IC_50_ values were performed using Student’s two-tailed t-test for paired or unpaired samples as appropriate.

## Results

*Plasmodium* spp. parasites were obtained from travellers returning to London from several malaria-endemic regions within Africa, between February and October 2012. Long-term culture was attempted on *P. falciparum* isolates from seven patients with microscopy-confirmed uncomplicated falciparum malaria, and who gave written informed consent for inclusion in the study. Seven *in vitro* parasite lines were successfully obtained, and each given a designation of the form HL12XX, for HTD / LSHTM 2012, plus a two-digit serial number. Also receiving HL designations were five non-falciparum parasite isolates (two *P. vivax*, one *P. ovale curtisi* and one *P. malariae*) collected for *ex vivo* drug sensitivity assays, which will be reported elsewhere.

### Patient travel histories and treatment

#### HL1204 - Kenya

This isolate was collected from a UK-resident adult male of Kenyan origin who had spent three weeks in Nairobi and coastal Kenya. Symptoms arose two weeks after return to the UK, and persisted for one week before attending HTD with 3.6% *P. falciparum* parasitaemia by microscopy. Schizonts and gametocytes were seen. Treatment was with intravenous artesunate due to high parasitaemia and early indicators of organ dysfunction. Parasitaemia estimates at 18, 45 and 68 hours were 3.2, 0.0001 and 0.001%, respectively, and the patient was discharged at ~72 hours on a full course of oral artemether-lumefantrine. The pre-treatment sample was placed into culture within 120 min of collection. An additional DNA preparation was obtained from the 18-hour sample.

#### HL1205 - Nigeria

The donor of this isolate was a UK-resident adult Nigerian woman who spent two weeks in Lagos with her husband. On return to the UK, symptoms arose, and the patient reported taking two tablets of Nivaquine (probably 200 mg chloroquine-sulphate) four days before presenting to UCLH with 3.2% peripheral parasitaemia, schizonts being present in the blood films. The patient received two doses of intravenous artesunate and, once improved, switched to a full oral course of artemether-lumefantrine. (The patient’s husband was also diagnosed with falciparum malaria, hospitalized and treated at the same time as his wife.) Estimates of peripheral parasitaemia at 15, 36 and ~50 hours were 0.38, 0.0001% and negative, respectively. There was a 16-hour delay between collection of the pre-treatment sample and initiation of culture. An additional DNA preparation was obtained from the 15-hour sample.

#### HL1209 – South Sudan

A UK-born UK resident who frequently travels to Africa, and with a previous history of three treated malaria infections, presented six days after return from a six-week trip to South Sudan. Microscopy confirmed 1.6% peripheral *P. falciparum* parasitaemia with schizonts present, and the patient was started on intravenous artesunate. Estimates of parasitaemia at 13, 37 and 57 hours were 3.2, 0.005% and negative, respectively. The patient was discharged on a full oral course of artemether-lumefantrine. There was a nine-hour delay between collection of the pre-treatment sample (at 00:45) and its processing for continuous culture. An additional DNA preparation was obtained from the 13-hour sample.

#### HL1210 – Ghana

This isolate was donated by an Indian-born young adult male, resident in Singapore but studying in the UK. The subject returned to the UK following a two-week trip to Ghana for a college project, and reported purchasing a course of anti-malarial chemoprophylaxis from a high street pharmacy in the UK before departure. Described as “two large tablets taken weekly and two small tablets taken daily”, this was almost certainly a course of chloroquine-proguanil, which does not require a prescription in the UK, but is no longer considered appropriate chemoprophylaxis for sub-Saharan Africa [41]. The patient stated that the course was not completed. He was well enough to attend a one-day international cricket match four days after returning to the UK, but developed symptoms the following day, which were managed with analgesics for three days. On the fourth day of symptoms, a peripheral *P. falciparum* parasitaemia of 0.3% was diagnosed at HTD, and the patient admitted to UCLH on oral artemether-lumefantrine. At 15 hours, parasitaemia had fallen to 0.05%, and the patient had improved sufficiently to recall the score in the cricket match. At 40 hours, parasitaemia was 0.0001% and the patient was discharged. The pre-treatment sample was collected at 17:45, and sat at ambient temperature in the laboratory overnight, before being placed into culture at ~10:00 next morning. An additional DNA preparation was obtained from the 15-hour sample.

#### HL1211 – Ghana

Blood samples from this patient, a young British adult male, were referred to HTD by an outside hospital in London, as a suspected case of artemether-lumefantrine treatment failure. The patient gave retrospective written signed consent to his physician for inclusion of parasite-positive samples in the study. Having presented with a *P. falciparum* parasitaemia of 0.03%, the patient was treated with oral artemether-lumefantrine, although it is unknown whether this was administered with fatty food, as recommended. At six hours, parasitaemia had increased to 4.2%, but fallen to 1.1% by 24 hours, and 0.001% by 48 hours. The patient was discharged following a negative blood film at 72 hours.

Fourteen days later, symptoms recurred, and *P. falciparum* parasitaemia of 0.7% was confirmed by the on-call microscopist at HTD, at 21:58 on a Sunday night. This EDTA vacutainer sample spent 60 hours at 4°C before being placed into continuous culture. The patient was treated with oral atovaquone-proguanil, and parasitaemia fell to 0.4 and then 0.07%, before eventual parasite clearance and a full recovery. An additional DNA preparation was obtained from the sample with 0.4% parasitaemia.

#### HL1212 – Nigeria

Blood samples for this isolate were referred to HTD from a hospital outside London because the patient, an adult UK resident female who recently travelled to Nigeria, carried an unusual hyper-gametocytaemia, in addition to asexual parasitaemia of approximately 1.5%. The patient’s immediate family gave written permission for any viable malaria parasites to be placed in culture for research. No post-treatment sample was available for analysis for this patient.

#### HL1214 – Burkina Faso

The donor of this isolate was a South African born adult female, a UK resident, who had spent three days in Burkina Faso on business. Malaria symptoms began 11 days after returning to London, and a blood sample taken at 21:46 on the 12th day was found to have 1.0% *P. falciparum* parasitaemia. The patient was treated with oral artemether-lumefantrine, and a single follow-up blood sample taken at 36 hours; this was negative and the patient was discharged to complete her oral chemotherapy at home. The pre-treatment blood sample was placed into continuous culture approximately 12 hours after collection. An additional DNA preparation was obtained from the 36-hour (microscopy negative) sample.

### Establishment of cultures and gametocyte production

The first parasite isolate (HL1204) was placed directly into standard growth medium of RPMI supplemented with 10% human AB serum. However, these parasites initially experienced a large decline in parasitaemia and took several weeks to recover their growth in culture. When the second isolate was received, the Methods for Malaria Research protocol was adopted [45] which combines a reduced amount of human serum (2%) with 0.5 g/L of Albumax II in the RPMI growth medium (see Methods for full details). In this growth medium, most parasite cultures adapted to *in vitro* culture without a marked decline in parasite growth. HL1204 was later also cultured in this alternative RPMI growth media. All the *P. falciparum* parasites received were successfully adapted to *in vitro* parasite growth after periods of time varying from 12 days to nine weeks, and initial stocks were frozen within approximately two weeks. Isolates were maintained in culture for no longer than 12 weeks after thawing in order to minimize the effects on parasite populations of genetic drift and loss of chromosome fragments.

It was noted that the first isolate, HL1204 from Kenya, produced gametocytes in culture spontaneously when the parasitaemia increased beyond 2%. Similarly, HL1205 was observed to produce scanty gametocytes at high parasitaemia *in vitro*. The gametocyte-producing property of the HL1204 isolate is currently being used to produce sexual parasite forms for transmission studies in mosquitoes (data not shown). Isolate HL1212 was obtained from a patient with peripheral hypergametocytaemia at the time of treatment, but after a few weeks of *in vitro* cultivation, there was no evidence of this phenotype persisting, as gametocytes were not observed in significant numbers in the established cultures under standard conditions.

### Multiplicity of infection (MoI)

Nested PCR amplification of the *P. falciparum* merozoite surface protein 1 (*pfmsp1*) or 2 (*pfmsp2*) locus was used to estimate the minimum number of clones present in the isolates as reported previously [39,46]. DNA samples for each isolate were obtained before drug treatment of the patients (Day 0) and one-day after drug treatment (Day 1) for comparison. A further sample was taken when the parasites were successfully adapted to grow in culture in order to examine whether the population of clones in the isolates had changed from the time of presentation at Day 0.

Aside from HL1214, the isolates were shown to contain at least four to six clones (Table 2). For the HL1214 isolate from Burkina Faso, a minimum multiplicity of two clones was estimated before drug treatment (Day 0) and both were found to persist into the culture adapted line. Similarly, three other isolates showed the same number of clones persisting into culture as those observed at presentation; HL1205 (four clones), HL1209 (six clones) and HL1210 (four clones). However, for isolates HL1204 and HL1212 there were fewer clones observed after culture adaptation than at presentation (Table 2).

**Table 2:** Multiplicity of infection in clinical isolates

**Isolate**	**Sample**	***pfmsp1***			***pfmsp2***		Minimum number of clones*	
		**K1**	**MAD20**	**RO33**	**FC27**	**IC**	**Per sample**	**Per isolate**
HL1204 Kenya	Day 0 pre-treatment	0	1	2	0	1	3	4
	Day 1 post-treatment	0	2	2	0	1	4	
	Culture-adapted	0	1	1	0	1	2	
HL1205 Nigeria	Day 0 pre-treatment	1	0	1	3	1	4	5
	Day 1 post-treatment	2	0	1	4	1	5	
	Culture-adapted	2	0	2	3	1	4	
HL1209 South Sudan	Day 0 pre-treatment	0	2	2	4	2	6	6
	Day 1 post-treatment	0	1	2	4	2	6	
	Culture-adapted	0	2	2	4	2	6	
HL1210 Ghana	Day 0 pre-treatment	2	2	0	0	1	4	4
	Day 1 post-treatment	2	2	0	0	1	4	
	Culture-adapted	2	2	0	0	1	4	
HL1211 Ghana	Day 0 pre-treatment	2	0	0	0	3	3	6
	Day 1 post-treatment	2	0	0	0	3	3	
	Culture-adapted	1	0	2	4	2	6	
^†^HL1212 Nigeria	Day 0 pre-treatment	3	2	1	2	1	6	6
	Culture-adapted	2	1	0	3	1	4	
HL1214 Burkina Faso	Day 0 pre-treatment	0	1	1	0	2	2	2
	Day 1 post-treatment	0	0	0	0	0	0	
	Culture-adapted	0	1	1	0	2	2	

*The minimum number of clones in each sample was estimated by adding the highest number of bands for each *Pfmsp* allele.

^†^No Day 1 post-treatment sample was obtained for HL1212.

Drug treatment had variable effects on the isolate populations. Thirty-six hours after drug treatment all parasites for HL1214 had been cleared below the level of detection in the PCR assay. However, all other isolates still had multiple clones persisting in the sample after the first 13–20 hours of drug treatment. For three isolates there was no change in the number of clones detected on Day 1 post-treatment; HL1209 (six clones), HL1210 (four clones) and HL1211 (three clones). For two isolates, HL1204 and HL1205, there was an additional clone detected *in vivo* at Day 1, as has been previously described for an imported malaria case [47].

### Sensitivity to anti-malarial drugs *in vitro*

The culture-adapted isolates were all tested for their sensitivity to a range of MMV-supplied anti-malarial agents using a standard 48-hour drug exposure and then measuring viability using the fluorescent PicoGreen method [38]. All the isolates were highly sensitive to the endoperoxides, dihydroartemisinin and OZ277, with IC_50_ values below 5 nM or 2 nM, respectively (Table 3). When examining the activity of the 4-aminoquinolines, it was observed that two of the isolates (HL1205 and HL1210) were resistant to chloroquine with IC_50_ values around 150 nM (chloroquine resistance defined as IC_50_ value >100 nM [48]). Interestingly, the histories of these two donors indicate that an incomplete course of chloroquine was probably taken, prior to presentation at HTD, in both cases.

**Table 3:** **Sensitivity of the *Plasmodium falciparum *patient isolates to a panel of anti-malarial compounds**

**Isolate**	**IC**_**50 **_**values (nM)**										
	**Endoperoxides**		**4-aminoquinolines**			**Arylaminoalcohols**			**Other**		
	**DHA**	**OZ277**	**CQ**	**AQ**	**PIP**	**MQ**	**LUM**	**QUI**	**PND**	**ATV**	**PYR**
HL1204 Kenya	2.7 ± 0.4	0.5 ± 0.1	13 ± 2	3.6 ± 0.5	27 ± 4	20 ± 2	119 ± 17	42 ± 10	2.6 ± 0.5	0.7 ± 0.1	>10000
HL1205 Nigeria	3.6 ± 0.7	0.5 ± 0.1	149 ± 10	5.6 ± 0.5	21 ± 4	17 ± 1	105 ± 20	58 ± 7	3.6 ± 0.9	0.7 ± 0.1	>10000
HL1209 South Sudan	4.7 ± 0.6	1.5 ± 0.5	12 ± 1	6.7 ± 1.1	26 ± 3	36 ± 6	110 ± 23	60 ± 7	2.2 ± 0.8	0.2 ± 0.03	>10000
HL1210 Ghana	1.7 ± 0.3	1.1 ± 0.2	150 ± 7	7.4 ± 0.5	16 ± 2	7.7 ± 0.8	24 ± 6	156 ± 17	1.9 ± 0.5	0.4 ± 0.1	>10000
HL1211 Ghana	4.1 ± 0.3	1.1 ± 0.2	12 ± 1	5.8 ± 0.8	31 ± 7	27 ± 3	67 ± 18	50 ± 16	1.7 ± 0.2	0.3 ± 0.04	>10000
HL1212 Nigeria	3.3 ± 0.8	0.6 ± 0.09	12 ± 3	4.2 ± 1.2	27 ± 4	23 ± 3	91 ± 21	22 ± 4	2.6 ± 0.9	0.8 ± 0.04	>10000
HL1214 Burkina Faso	3.3 ± 0.7	0.6 ± 0.07	14 ± 3	4.2 ± 0.3	23 ± 7	23 ± 1	77 ± 28	52 ± 10	1.3 ± 0.6	0.7 ± 0.2	62 ± 25
**Laboratory strains**											
3D7 Netherlands	5.5 ± 1.0	1.1 ± 0.3	15 ± 2	7.6 ± 1.7	14 ± 1	23 ± 3	73 ± 22	53 ± 6	5.0 ± 0.9	0.3 ± 0.2	28 ± 7
K1 Thailand	5.2 ± 0.9	0.8 ± 0.3	374 ± 55	10 ± 3	37 ± 3	6.8 ± 2.9	32 ± 4	289 ± 40	16 ± 5	4.3 ± 1.0	>10000

The abbreviations for the antimalarial compounds are: dihydroartemisinin (DHA), arterolane, synthetic endoperoxide (OZ277), chloroquine (CQ), amodiaquine (AQ), piperaquine (PIP), mefloquine (MQ), lumefantrine (LUM), quinine (QUI), pyronaridine (PND), atovaquone (ATV) and pyrimethamine (PYR).

The IC_50_ values are averaged from at least three independent experiments with some repeated on more than ten occasions. All data are presented as mean ± S.E.M. In several instances, the IC_50_ values for pyrimethamine exceeded the limits of detection for the concentration range adopted in the experiments and are thus reported as >10,000 nM.

The five remaining isolates were highly sensitive to chloroquine (<15 nM) and all seven isolates were sensitive to amodiaquine and piperaquine with IC_50_ values below 8 nM or 32 nM, respectively (Table 3). Sensitivity to desethyl-amodiaquine was not evaluated. Among the arylaminoalcohol class of compounds HL1210 was the most sensitive of the isolates to both mefloquine (7.7 nM) and lumefantrine (24 nM). The IC_50_ values for mefloquine in the remaining isolates ranged from 17–36 nM. The cut-off for mefloquine resistance has been proposed to be 30 nM [48] and thus the HL1209 isolate could be considered resistant by this definition. However, many of the isolates demonstrate a reduced sensitivity to mefloquine with IC_50_ values above 20 nM. In contrast to the results obtained for the other arylaminoalcohol compounds, isolate HL1210 was the least sensitive to quinine (156 nM) with the IC_50_ values for the remaining isolates all below 60 nM (Table 3). For the remaining compounds, all seven isolates were sensitive to pyronaridine, with IC_50_ values below 4 nM, and all highly sensitive to atovaquone with IC_50_ values below 1 nM (Table 3). Lastly, all but one of the isolates (i e, HL1214, IC_50_ value = 62 nM) were highly resistant to the dihydrofolate reductase (DHFR) inhibitor pyrimethamine with IC_50_ values greater than 10 μM (pyrimethamine resistance defined as an IC_50_ value > 100 nM [48]).

Of particular interest are *in vitro* responses to lumefantrine, as almost all of the contributing patients received a course of oral artemether-lumefantrine as part of their treatment, and one isolate, HL1211, was obtained from a donor that appeared to have failed treatment on this combination. Apart from HL1210, all isolates exhibited quite low *in vitro* sensitivity to lumefantrine with IC_50_ values ranging from 67–119 nM, with the culture-adapted line from isolate HL1211 in the middle of this range at 67 nM. This line took many weeks to recover to sustainable levels *in vitro*, and the clonal data in Table 2 suggests that very minor genotypes undetectable in the pre-treatment sample, which was the apparent in *vivo* treatment failure from the earlier course of artemether-lumefantrine, gradually recovered in abundance to predominate in the adapted line. Separation of the sub-clones in isolate HL1211 may vary in intrinsic lumefantrine sensitivities, which is “averaged” among them in the bulk multiclonal culture, thus obscuring any significant lumefantrine resistant phenotype. It may also be that *in vitro* estimation of lumefantrine sensitivity is a poor correlate of *in vivo* treatment response, and to date no validated IC_50_ cut-off for adequate lumefantrine sensitivity has been established; all of the contributing patients apart from HL1211 recovered well on artemether-lumefantrine combination therapy, as either sole therapy, or as a follow-on oral regimen after intravenous artesunate.

### Genes associated with drug resistance

#### Pfcrt

Multiplex real-time PCR was used to identify the alleles at the chloroquine resistance transporter (*pfcrt*) codons 72–76 for all the isolates with samples taken on Day 0 (pre-treatment), Day 1 (post-treatment) and after culture-adaptation (Table 4). For both the HL1211 and HL1214 isolates, the wild type CVMNK was the only detectable haplotype in all samples. This haplotype is associated with sensitivity to chloroquine [6] and supports the low chloroquine IC_50_ values reported for these isolates in Table 3. In the HL1205 and HL1210 isolates, which both exhibited high chloroquine IC_50_ values, only the chloroquine-resistant CVIET haplotype was detected for all samples; as noted above, both isolates are from patients who reported self-medication with tablets that were almost certainly chloroquine. The remaining isolates, HL1204, HL1209 and HL1212 all show a mixed CVMNK/CVIET haplotype on Day 0 and Day 1 (no data for HL1212 on Day 1). However, when these isolates are adapted to grow in culture, only the CQS CVMNK haplotypes are detected. No SVMNT haplotypes, associated with resistance to amodiaquine and chloroquine, were observed in any of the isolates.

**Table 4:** Haplotypes of parasite markers associated with drug resistance

**Isolate**	**Sample**	**Haplotype**				
		***Pfcrt***	***Pfmdr1***	***Pfdhps***	***Pfdhfr***	***Pfmdr1***
		**72-76**	**86, 184, 1034, 1042, 1246**	**431, 436, 437, 540, 581, 613**	**16, 50, 51, 59, 108, 140, 164**	**Gene copy number**
	Wild type reference	**CVMNK**	**NYSND**	**ISAKAA**	**ACNCSVI**	
HL1204 Kenya	Day 0 pre-treatment	**CVMNK** / CVIET	NFSND	ISGEAA	ACNRNVI	1
	Day 1 post-treatment	**CVMNK** / CVIET	NFSND	ISGEAA	ACNRNVI	1
	Culture-adapted	**CVMNK**	NFSND	ISGEAA	ACNRNVI	1
HL1205 Nigeria	Day 0 pre-treatment	CVIET	**NYSND**	IFAKAS	ACIRNVI	1
	Day 1 post-treatment	CVIET	**NYSND**	IFAKAS / ISGKAA	ACIRNVI	1
	Culture-adapted	CVIET	**NYSND**	IFAKAS	ACIRNVI	1
HL1209 South Sudan	Day 0 pre-treatment	**CVMNK** / CVIET	NFSND	**ISAKAA**	ACICNVI	1
	Day 1 post-treatment	**CVMNK** / CVIET	NFSND	**ISAKAA**	ACICNVI	1
	Culture-adapted	**CVMNK**	NFSND	**ISAKAA**	ACICNVI	1
HL1210 Ghana	Day 0 pre-treatment	CVIET	YFSND	ISGKAA	ACNRNVI / ACIRNVI	1
	Day 1 post-treatment	CVIET	YFSND	ISGKAA	ACNRNVI / ACIRNVI	1
	Culture-adapted	CVIET	YFSND	ISGKAA	ACNRNVI / ACIRNVI	1
HL1211 Ghana	Day 0 pre-treatment	**CVMNK**	**NYSND**	IAGKAA	ACIRNVI	1
	Day 1 post-treatment	**CVMNK**	**NYSND**	IAGKAA	ACIRNVI	1
	Culture-adapted	**CVMNK**	NFSND	**ISAKAA**	ACICNVI	1
*HL1212 Nigeria	Day 0 pre-treatment	**CVMNK** / CVIET	**NYSND** / NFSND	ISGKAA	ACIRNVI	1
	Culture-adapted	**CVMNK**	NFSND	ISGKAA	ACIRNVI	1
^§^HL1214 Burkina Faso	Day 0 pre-treatment	**CVMNK**	NFSND	ISGKAA	**ACNCSVI** / ACNRNVI	1
	Culture-adapted	**CVMNK**	NFSND	ISGKAA	**ACNCSVI** / ACNRNVI	1

*No Day 1 post-treatment sample was obtained for HL1212.

^§^No haplotype data was obtained for the Day 1 post-treatment sample most likely due to complete parasite clearance shortly after drug administration.

For clarity, wild type haplotypes for each gene reported are highlighted in bold.

#### Pfmdr1

The isolates were sequenced to look for polymorphisms in the multidrug resistance gene (*pfmdr1*) associated with drug resistance: namely, N86Y, Y184F, S1034C, N1042D and D1246Y. No changes were observed from the wild type codons S1034, N1042 and D1246 for any of the isolate samples examined (Table 4). All but one isolate (HL1210; 86Y) harboured the wild type Asn at codon 86. The HL1212 isolate was the only isolate to harbour a mixed haplotype in the pre-treatment sample but only a single haplotype was evident in the HL1212 culture-adapted sample. Real-time PCR analysis of each the samples revealed all the isolates possess only a single copy of the *pfmdr1* gene (Table 4).

#### *Pfdhps* and *Pfdhfr*

The isolates were also examined for polymorphisms in the genes associated with resistance to the antifolate drugs. Mutations in the dihydropteroate synthase gene, *pfdhps*, are responsible for resistance to the anti-malarial sulphadoxine. Important amino acid changes associated with resistance include I431V, S436A, A437G, K540E, A581G and A613S. Only one isolate, HL1209, encoded the wild type amino acids at all six positions in *pfdhps* (Table 4). Three isolates (i e, HL1210, HL1212 and HL1214) showed a single amino acid change, A437G, while two of the double mutants contained this mutation plus either a K540E (for HL1204) or S436A (for HL1211). Isolate HL1205 was unusual as its two amino acid changes did not include the A437G mutation but instead harboured the mutations S436F and A613S. Furthermore, HL1205 also showed a mixed haplotype in its post-treatment sample, which did include the single mutant A437G (Table 4).

Resistance to pyrimethamine is caused by mutations in the dihydrofolate reductase gene, *pfdhfr*, and is associated with the following amino acid changes: A16V, N51I, C59R, S108N and I164L. The only isolate carrying the pyrimethamine sensitive wild type haplotype was HL1214 (Table 4). All the other isolates harboured pyrimethamine resistance haplotypes and all contained the amino acid change from a serine to asparigine in codon 108 (S108N) which has been suggested to be a vital first step in the development of mutants with higher levels of pyrimethamine resistance [49]. The double mutants carried an additional mutation, changing either an asparigine to isoleucine at codon 51 (N51I – HL1209) or a cysteine to arginine at codon 59 (C59R –HL1204, HL1210, HL1214). The triple mutants contained mutations in all three aforementioned codons (i e, N51I, C59R and S108N). No mutations were observed in the amino acids at codons 16, 50, 140 and 146, which have also been implicated in resistance to pyrimethamine and/or cycloguanil [43,50]. Interestingly, within each isolate, none of the *pfdhfr* haplotypes varied among the pre-treatment, post-treatment or culture-adapted samples.

#### Pfap2-mu

Sequences for this gene were determined for all available *ex vivo* and *in vitro* parasite preparations. None of the previously described non-synonymous polymorphisms at codons 146, 160, 199, 233 or 437 of *pfap2mu* were found in any parasite line, or in any *ex vivo* parasite DNA sample. Some variation was observed in the previously described poly-Asn tracts which occur between codons 226 and 234, and between codons 326 and 331 [36]. A synonymous mutation from A to G at the third position of codon 163 (Glu) was present in HL1209, HL1211 and HL1214, usually as a mixture of both alleles. However, in HL1211 all *ex vivo* parasite isolates carried the GAA form of codon 163, whereas the culture-adapted parasite line appeared to harbour only GAG alleles.

## Discussion

Using the Methods in Malaria Research protocol for culturing new isolates [45], it was possible to successfully adapt all seven of the African *P. falciparum* patient isolates to *in vitro* conditions. The parasites grew more readily in the combination of 0.5% w/v Albumax II with 2% human AB serum when compared with the HL1204 line initially grown in 10% human AB serum in the absence of Albumax II.

The African isolates in this study were shown to be composed of a complex mixture of parasites with a minimum of between two and six clones. This level of complexity is similar to that reported by Robinson *et al.*[46]. In that study also, patients presenting with malaria to HTD after travel to Africa were assessed for MOI and found to harbour complex mixtures of between three and five clones using both *msp1* and *msp2* genotyping and whole genome analysis [46]. Differences in *pfmsp1* and *pfmsp2* repertoires were noted between *ex vivo* isolates analysed directly, and those from cultures established from the same sample over time, most notably for HL1211 (Table 2). This can be explained by the stochastic variation seen in PCR amplification of complex mixtures [50], and the fact that *ex vivo* PCR was performed on the equivalent of 10 μL of peripheral blood, whereas at least 100 times that volume of blood was used to establish each long-term culture, and this could easily have harboured additional genotypes. Examining clonality through PCR amplification of the *msp1* and *msp2* loci is subject to error and can only provide a minimum estimate of MOI [51]. How then does this observed genetic complexity arise? Polyclonality need not arise from multiple mosquito bites and can result from a single sporozoite inoculation, as suggested by studies of infections in travellers with minimal exposure to infective bites [46,47]. This genetic complexity in African isolates represents an important difference from those obtained from other regions, thereby emphasizing the potential benefit of establishing multiclonal isolates for *in vitro* studies as a more accurate model of the African *P. falciparum* infections found in the field. When the culture-adapted parasites were compared with the original “pre-treatment” source parasites it was found that in four of the seven isolates, the number of clones detected remained unchanged after adaptation. Despite the loss of one and two clones in culture-adapted HL1204 and HL1212, respectively, the data in Table 2 suggest that the culture conditions adopted are favourable to support the growth of multiple clones and that these isolates should therefore maintain their genetic complexity over time.

All the patients in this study were treated with ACT therapy which consists of a rapid-acting artemisinin-based compound (artemether) co-formulated with a longer-acting compound (lumefantrine), although in two cases this oral regimen followed initial treatment with intravenous artesunate. In only one of the patients (the donor of HL1214) were the parasites cleared completely within 24 hours of the treatment administration below the detection level of the nested PCR assay. In the remaining isolates multiple clones remained detectable after treatment, and in two of the samples (HL1204, HL1205) the number of parasite clones detected had increased by one compared with the number observed pre-treatment. Such complicated clonal dynamics during treatment do not imply that parasites are resistant to anti-malarial agents, but are due to fluctuations in relative clonal densities which frequently occur in treated patients [47]. Clones present in the pre-treatment sample at low density relative to other well-represented clones in the overall parasite population may not be amplified sufficiently to enable detection. However, one day post-treatment, with the parasite load decreased after the drug administration, these clones may become more evident using the nested PCR method.

### Drug sensitivity/resistance

The WHO-recommended combination therapy approach was intended to prevent parasites from developing resistance to the rapid-acting, highly potent but quickly eliminated artemisinin derivatives. All the isolates in this study were shown to be highly sensitive to dihydroartemisinin and the synthetic endoperoxide, OZ277. Recently, reports have emerged of artemisinin resistance characterized by a “delayed clearance” phenotype in Thailand, Cambodia and Myanmar [28,30-34]. A conventional 48-hour drug exposure assay was used to assess parasite growth in this study. Under similar *in vitro* assay conditions, previous studies have been unable to detect artemisinin tolerant parasites, and new assays have been proposed that use a short pulse exposure to the artemisinin derivative DHA [34,52]. Although it is of interest to screen the isolates presented here with such assays, a “delayed clearance” phenotype similar to that seen in Southeast Asia has not yet been reported in African studies of ACT efficacy in the field.

Most of the culture-adapted isolates (five out of seven) were sensitive to chloroquine (Table 3), and all five harboured the CVMNK haplotype at codons 72–76 of *pfcrt* (Table 4). A recent study has shown that treatment with the ACT artemether-lumefantrine (Coartem^®^) leads to the selection of parasites with the lysine at codon 76 in Tanzanian parasites [53]. The two CQR isolates were both confirmed to harbour the CVIET haplotype commonly found in Africa and Asia [54]. In countries where amodiaquine was used as first-line monotherapy for malaria, the predominant CQR haplotype emerging has been SVMNT [55]. In Africa however, chloroquine was preferred to amodiaquine and, not surprisingly, the SVMNT CQR haplotype found mostly in Asia, South America and Papua New Guinea was not observed in these African isolates. Although the CVIET haplotype in the mixed CVMNK/CVIET pre-treatment isolates (i e, HL1204, HL1209 and HL1212: Table 4) was not detected in the culture-adapted isolates, these clones may still be present but below the level of sensitivity of the assay. Using chloroquine drug pressure it may be possible to select for these minor clones *in vitro* in the culture-adapted lines. The reason for these CQR clones not thriving from the mixed CVMNK/CVIET pre-treatment parasites into culture adaptation may be related to a fitness cost associated with mutations in the CQR genes, *pfcrt* and *pfmdr1*[56].

Mutations in the *pfmdr1* gene have been implicated in modulating the sensitivity to several classes of anti-malarial agents including the 4-aminoquinolines, arylaminoalcohols and endoperoxides [57-59]. Three haplotypes representing the five codons implicated previously in drug resistance (i e, 86, 184, 1034, 1042, and 1246) were identified among the HL isolates. These were NYSND (wild type), NFSND and YFSND. While sensitivity to chloroquine has been shown to be mediated by mutations in both *pfcrt* and *pfmdr1*[6,7,58], the chloroquine IC_50_ values reported here do not correlate with any particular *pfmdr1* haplotype. Examining the *pfcrt* haplotype in these isolates is sufficient to predict accurately the chloroquine sensitivity for these isolates irrespective of changes in *pfmdr1*.

As with chloroquine, sensitivity to the anti-malarial quinine has been shown to be modulated by mutations in both the *pfcrt* and *pfmdr1* genes [58-60]. In contrast to chloroquine though, the sensitivity to quinine in these isolates cannot be explained by its *pfcrt* haplotype alone. On the other hand, the one isolate showing a reduced sensitivity to quinine (HL1210; IC_50_ value ≥2.5-fold relative to all other isolates) was shown to be the only one harbouring the YFSND haplotype in *pfmdr1*. This haplotype differs from the two other *pfmdr1* haplotypes observed in the isolates by the presence of Tyr encoded at codon 86. Similarly, the YFSND haplotype found in HL1210 results in the greatest sensitivity to mefloquine and lumefantrine. All the remaining isolates with the Asn at codon 86 were less sensitive to mefloquine and lumefantrine (all >2-fold IC_50_ value relative to HL1210). The prominence of the Asn observed at codon 86 in these isolates is not surprising. A previous *in vitro* study showed that parasites of this genotype were less sensitive to arylaminoalcohol compounds (e g, mefloquine, lumefantrine) and endoperoxides (e g, artemisinin) [57]. This might imply that in order to evade the toxicity of these compounds *in vivo*, parasites carrying the asparigine at codon 86 would be selected in place of those carrying the tyrosine. Indeed, several studies have confirmed this concerning trend. In several African countries where Coartem^®^ has been used as first-line treatment for uncomplicated *P. falciparum* malaria, reports have emerged that the N86 codon of *pfmdr1* is being selected [35,42,61,62]. Furthermore, Mwai *et al.* reported that parasites harbouring both the K76 in *pfcrt* and N86 in *pfmdr1* had higher lumefantrine IC_50_ values than those with mutant alleles [63]. This N86 selection then could explain the high mefloquine and lumefantrine IC_50_ values reported in these isolates while the Y86 found in HL1210 is consistent with sensitivity to these compounds.

Multiple copies of the *pfmdr1* gene have been linked to reduced susceptibility to lumefantrine *in vitro* in Thai isolates [64]. However, in the limited number of African isolates reported here, no increases in *pfmdr1* copy number were observed irrespective of their susceptibility to lumefantrine. This does not rule out a role for *pfmdr1* copy number in reduced drug susceptibility in Africa although as it has been observed recently in Sudanese malaria patients that *pfmdr1* amplification was linked to recurrent infection after treatment with artemether-lumefantrine [35]. For the *pfap2mu* locus, only minor variation in the length of Asn-rich tracts was observed in the isolates examined. In HL1211 an allele with a variant codon 163 appeared to be selected in the established culture line, but this is a synonymous change and is unlikely to have any direct bearing on drug response.

Pyronaridine, a compound currently in Phase IV clinical trials as a licensed combination therapy with artesunate (Pyramax^®^), is highly potent against all isolates in this study irrespective of their resistance to chloroquine and pyrimethamine and altered sensitivity to the arylaminoalcohol compounds. Previous studies have proposed that CQR strains exhibit cross-resistance to pyronaridine [65] but recent evidence suggests that pyronaridine remains effective against CQR strains [66]. The results from the small sample of isolates provided here suggest that pyronaridine would be an effective treatment against multiclonal African parasites of diverse origin and drug-resistance profiles. Furthermore, the naphthoquinone atovaquone which is marketed as a fixed combination with proguanil (Malarone), and widely used for chemoprophylaxis by travellers to malaria-endemic regions, was highly potent against all the isolates tested in this study.

The high degree of resistance to the dihydrofolate reductase inhibitor pyrimethamine in all but one of the isolates (i e, HL1214) probably reflects the widespread use of the antifolate therapy Fansidar (sulphadoxine/pyrimethamine) as first-line anti-malarial treatment across Africa for many years preceding the WHO recommendation for using ACT as first-line treatment. The IC_50_ values presented for pyrimethamine (Table 3) are supported by the DHFR haplotypes reported for each isolate in Table 4. As predicted from its sensitivity to pyrimethamine (62 nM; Table 3), HL1214 was the only isolate with the wild type 3D7-like haplotype. Interestingly, a resistant haplotype was also detected for this isolate suggesting that these parasites are present probably as a minor population within the isolate given that there was no effect on increasing the IC_50_ value beyond the 100 nM cut-off for resistance. All of the other resistant isolates contained either a double or triple mutation in *pfdhfr* that would be expected to result in a “moderate” or “high” level of pyrimethamine resistance, respectively [48,49,67]. All the resistant isolates harboured the change from a serine to asparigine in codon 108 (S108N) which has been suggested to be a critical first step in the development of mutants with higher levels of pyrimethamine resistance [49]. The double mutants carried an additional mutation, changing either an asparigine to isoleucine at codon 51 (N51I – eg., HL1209) or a cysteine to arginine at codon 59 (C59R – e g, HL1204, HL1210, HL1214) which are expected to result in moderate resistance to pyrimethamine, whereas parasites with mutations at all three codons (i e, N51I, C59R and S108N) are expected to display high level pyrimethamine resistance [49]. No mutations were observed in the amino acids at positions 16, 50, 140 and 164, which have also been implicated in resistance to pyrimethamine and/or cycloguanil [43,49].

The anti-malarial agent sulphadoxine, a drug used in combination with pyrimethamine in the drug treatment Fansidar, was not included in the panel of compounds tested in this study. However, the dihydropteroate synthase gene (*pfdhps*) was sequenced to look for mutations associated with sulphadoxine resistance. All but one (i e, HL1209) of the isolates contained parasites with the A437G amino acid change. This polymorphism, alone or in combination with other mutations in *pfdhps*, predominates in resistant field isolates [68]. When the A437G mutant is combined with the K540E mutation, this double mutant is strongly associated with clinical Fansidar treatment failure [69]. Interestingly, the HL1209 isolate harbours the “sulphadoxine sensitive” *pfdhps* haplotype (ISAKAA) but was shown to be carry resistant alleles for *pfdhfr*. It has been proposed previously that mutations in *pfdhps* occur only once the parasites in the population carry at least a double mutant allele in *pfdhfr*[68]. Quintuple mutants with two mutations in *pfdhps* (A437G, K540E) and three mutations in *pfdhfr* (N51I, C59R, S108N) are strongly associated with Fansidar treatment failure [69]. Isolates HL1205 and HL1211 both carry the requisite triple mutations in *pfdhfr* and harbour double mutations in *pfdhps* but these double mutations in *pfdhps* differ from those previously linked with treatment failure. It is unclear whether these quintuple mutant variants are as likely to result in clinical failure after Fansidar treatment.

## Conclusions

This study describes the establishment in continuous culture, *in vitro* drug sensitivity testing and molecular characterization of a series of multiclonal *P. falciparum* isolates taken directly from UK malaria patients following recent travel to various malaria-endemic countries in Africa. These “HL” isolates are available as an open resource for studies of drug response, antigenic diversity and other aspects of parasite biology.

## Competing interests

The authors declare that they have no competing interests.

## Authors’ contributions

CJS and DAvS conceived and designed the study, with input from RB and XCD. CJS, CH, SGW and PLC interviewed patients and collated clinical data. RB, DAvS, GH, KBB and NG performed the experiments. XCD supplied the anti-malarial compounds and provided comments on the manuscript. DAvS, RB and CJS analysed the data and wrote the paper. All authors read and approved the final manuscript.

## Supplementary Material

Additional file 1 Primer sequences and annealing conditions for candidate gene amplification. Click here for file
