# Supplementary material for: Culture-adapted Plasmodium falciparum isolates from UK travellers: in vitro drug sensitivity, clonality and drug resistance markers
Source: Malar J. 2013 Sep 13;12:320. doi: 10.1186/1475-2875-12-320 (PMC3847303; doi:10.1186/1475-2875-12-320)
Supplement: Additional file 1 — Primer sequences and annealing conditions for candidate gene amplification. [file 1475-2875-12-320-S1.docx]

### Additional File 1: Primer sequences and annealing conditions for candidate gene amplification

| **Gene** | **Fragment** | **1^st^ amplification primers (5’ to 3’)** | **Size^a^** | | **T_a_^b^** | **2^nd^ amplification primers (5’ to 3’)** | **Size** | **T_a_** |
| --- | --- | --- | --- | --- | --- | --- | --- | --- |
| *pfmdr1* | 1 | FN1 & REV/C1 (ref. 24) | 578 bp | 50°C | | MDR2/1 & NEWREV1 (ref. 24) | 534 bp | 60°C |
|  | 2 | newfr2_F: CAGGAAGCATTTTATAATATGC  newfr2_N1R: GCAGCAAACTTACTAACACGTT | 908 bp | 55°C | | newfr2_F  newfr2_N2R: TTCCAATGTTGCATCTTCTCT | 879 bp | 55°C |
| *pfap2-mu* | 1 | Fw - AAGACTGTCAAATGTAAAAGACCC  Rev - CTCATGTAAAACAAAAAGTGAGG | 2247 bp | | 50°C | Fw - GTTAACACGATTAGCGTCATTTG | 578 bp | 53°C |
|  |  |  |  |  |  | Rev - GTCCTATTATGTATATGTGGATC |  |  |
|  | 2 |  |  |  |  | Fw - GATATCCACAAACATTAGAAGTG | 841 bp | 52°C |
|  |  |  |  |  |  | Rev - CCATCTGGTGGTGTGAAGG |  |  |
|  | 3 |  |  |  |  | Fw - GCATATTTCATCATTGTGTTACC | 753 bp | 53°C |
|  |  |  |  |  |  | Rev - ACACCCGATTGAACTATTTATAC |  |  |

T_a_ : annealing temperature
